# 

**Published:** 1886-01

**Authors:** 


### INDEX TO VOLUME XL.

Association Work.................... 5
Address—C. H. James............. 10
Additional Thoughts on Pyorrohea Alveolaris............... 57
Aesthetic Application of Dental Art.. ................................ 72
Atropia in Acute Coryza......... 93
Association.............................. 105
A Bill to Secure the Better Education of Practitioners of Dental Surgery, etc........... 122
American Dental Association... 154
A Reliable Remedy for Ozena. 140
A Remarkable Instance of Longevity........................ 141
A Question of Sanity............... 150
Alabama State Dental Society... 160
Abstract of a Lecture on Comparative Odontology.......... 226
American Medical Association.. 263
Anaesthetics............................ 309
A Rational Conclusion............ 311
Address — Geo. J. Friedrichs, M.D., D.D.S..................... 328
Alabama State Dental Association ..... .............................. 337
Address on Dental and Oral Surgery ............................ 377
Atrophic Progressive Myopathy 418
American Dental Association ..................................476, 425
Aluminium Bronze in Medicine and Dentistry................... 508
A Tooth Growing in the Nose... 512
Bibliographical..........159, 529, 580
Bridge Work............................ 231
Beware of Bogus Dentists ...... 312
Board of Examiners................ 320
Biographical........................... 582
Cocaine................................... 42
Chicago Dental Society............ 152
Central Illinois Dental Society.. 478
Chancre of the Gum................ 573
Choaking................................ 257
Dental Spiritualism................. 57
Deutsche Monatschchrift for Zahnheilkunde................ 144
Death from Lodgment of a Gold Plate in the Bronchial Tubes.................................. 184
Dr. E. F. Semple..................... 214
Dentition of Early American Races ................................ 252
Distinguished Honor............... 370
Dental Erosion in Scrofula...... 420
Death of Dr. C. Robin............. 420
Deafness Cured by Excision of Tonsils ............................ 503
Diseases Peculiar to Bakers...... 512
Dental Education..................... 537
Drunkard's Epilepsy............... 575
Editorial Change..................... 319
Esmarche's Bandage in Anæsthesia.................................. 619
Every Day Practice...........321, 325
Forty-Second Annual Meeting of the Mississippi Valley Association of Dental Surgeons.......................... 186
Faith Healing.......................... 303
Geographical Distribution of Dental Caries.................. 14
Gas Furnaces vs. Coal or Coke.. 70
Graduates in Dental Surgery for the Year 1886................... 356
Georgia State Dental Society... 404
Herbst's Method..................... 47
" " A Criticism of 594
Hydronaphthal—A New Antiseptic.................................. 83
Hypodermic Medication.......... 92
Heredity......... ...................... 109
Hydrogen Peroxide and its Utility.................................. 179
International Medical Congress. 50
Items of Interest about the Pennsylvania Hospital...... 95
Illinois State Dental Society.126, 213
Indigestion and its Treatment... 133
Indigestion in Children........... 136

Is Cancer Hereditary ?........... 146
Injections of Osmium Acid in Neuralga ........................ 310
International Congress......316, 318
In Memory of Dr. W. Hart Cameron.......................... 372
Is Dentistry a Profession ?...... 491
Jealousy Among Doctors......... 140
Kansas State Dental Association.................................. 214
Local Treatment of Ranula...... 89
Living with a Bullet in Her Brain.............................. 94
Louisiana State Dental Society. ..............................107, 267
Midland Railway.................... 54
Menthol—A Local Anæsthetic.. 96
Mississippi Valley Association of Dental Surgeons........... 195
Michigan State Dental Society.. 212
Manicure.............................. 308
Mechanical Dentistry.............. 401
Nervousness Hereditary and Acquired........................ 19
Ninth International Medical Congress......................95, 104
Napelline in the Treatment of Odontalgia....................... 143
No Right to Trifle with Human Life................................ 255
Northern Ohio Dental Association..................................... 291
National Dental Association..... 320
National Association of Dental Faculties ....................... 505
National Association of Dental Examiners...................... 508
Ohio State Dental Society......... ...................26, 528, 579, 583
Ohio State Dental Society, Minutes of............................ 621
Obituary..............54, 214, 264, 630
Observation on the Oleates....... 138
Oral Diseases........................ 215
Oral Diseases and Caseous Phthisis .......................... 616
Oil of Sassafras for Neuralgia... 256
Pyorrhœa Alveolaris Infection.. 29
Prevention of Bad Teeth......... 90
Peculiar Effect of Corrosive Sublimate........................ 91
Preserving Cocaine Solutions.... 139
Pilocarpin in Toothache.......... 150
Probable or Possible Poisoning from Dentist's Amalgam.... 161
Physiology and Pathology of First Dentition................. 373
Post Graduate Study............... 479
Proper Care of Poor Children's Teeth.................................. 498
Proceedings Ohio State Dental Society ........................... 583
Purity of Drinking Water...515, 575
Reminescences........................ 67
Riggs's Disease...................... 78
Salmagundi..44, 97, 155, 200, 213, 257, 364, 421, 474, 516, 576, 626
Salicylate of Cocaine in the Treatment of Trigeminal Neuralgia.......................... 143
Scrivener's Palsy.................... 610
Some Phenomena Observed in the Use of Antiseptics in Dental Surgery................. 175
Summary of Discussions.......... 243
Small Pox and Vaccination...... 255
Society Meetings.................... 366
Syphilis in its Relation to Dental and Oral Surgery... 388
Southern Dental Association... 477
Southern Dental Association..... 540
Treatment of Third Molars...... 24
The Etiology of Rachitis......... 128
Therapeutical Hints............... 142
Treatment of Pulpless Teeth.168 171
The American Dental Ass'n..... 206
Third Molar, Eruption of........ 583
To State and Local Dental Societies ............................ 262
The Finger Nails in Disease..... 306
The Treatment of Pulpless and Gaseous Teeth.................. 326
The Wear and Tear of Modern Life.................................. 415
The Influence of Beer, Tobacco, etc. upon Digestion............ 511
Two Cases of Swallowing False Teeth.............................. 513
The Spirit of Imitation in Medicine.................................. 513
Thinking and Working............ 514
The International Medical Congress................................. 527
Virginia State Dental Assoc'n... 477
Where shall the American Dental Association Meet in 1886?............................. 209
Where to Meet....................... 211
Zymosis................................ 537

### CONTENTS.

COMMUNICATIONS.

Association Work ... 5
Address ... 10
Geographical Distribution of Dental Caries ... 14
Nervousness, Hereditary and Acquired ... 19
Treatment of Third Molars ... 24

PROCEEDINGS.

Ohio State Dental Society ... 26

Additional Thoughts on Pyorrhœa Alveolaris ... 27

Pyorrhœa Alveolaris Infection ... 29

The Eruption of the Permanent Teeth ... 39

CORRESPONDENCE.

Cocaine ... 42

SALMAGUNDI ... 44

EDITORIAL.

Herbst's Method ... 47

International Medical Congress ... 50

Midland Railway ... 54

Obituary ... 54

### THE DENTAL REGISTER,

A MONTHLY MAGAZINE DEVOTED TO THE INTERESTS OF THE

### DENTAL PROFESSION.

*Terms Reduced to* $2.00 *Per Year.* *Single Copies,* 25 *Cents.*

### EDITED BY PROF. J. TAFT, D.D.S.

—AND—

### Published by SAM'L A. CROCKER & CO., Ohio Dental and Surgical Depot.

The volume commences in January and closes in December.
The REGISTER being issued monthly is always fresh, therefore Indispensable to the practitioner who desires to keep posted up with the new inventions and improvements which are daily developed. Neither expense nor effort will be spared to give value and variety to its contents. Articles will be illustrated with well executed engravings whenever they will add to the interest or assist to explanation.
Its extensive circulation and frequency of issue make it one of the BEST DENTAL ADVERTISING MEDIUMS in the United States.
All subscriptions must begin with January or July.
Local or special notices, five lines or less, will be inserted for $3 each insertion; over five lines, 50 cts. per line.
All advertising bills payable in advance, or at furthest, after the issue of the first number containing the advertisement. All transient advertisements to be paid for in advance.
☞CONTRIBUTIONS SOLICITED.
Exclusively Editorial Communications should be addressed to the Editor.
The latest number of the REGISTER may be ordered with or without other goods at any time, and all letters should be addressed to

**SAM'L A. CROCKER & CO., Ohio Dental and Surgical Depot, Cincinnati, O.**

